# Co‐administration of 2’3’-cGAMP STING activator and CpG-C adjuvants with a mutated form of HPV 16 E7 protein leads to tumor growth inhibition in the mouse model

**DOI:** 10.1186/s13027-021-00346-7

**Published:** 2021-01-26

**Authors:** Fariba Dorostkar, Arash Arashkia, Farzin Roohvand, Zabihollah Shoja, Mohsen Navari, Maryam Mashhadi Abolghasem Shirazi, Zahra Shahosseini, Mohammad Farahmand, Mohammad Sadegh Shams nosrati, Somayeh Jalilvand

**Affiliations:** 1 Department of Virology, School of Public Health, Tehran University of Medical Sciences, 14155 Tehran, Iran; 2 Department of Molecular Virology, Pasteur Institute of Iran, Tehran, Iran; 3 Department of Medical Biotechnology, School of Paramedical Sciences, Torbat Heydariyeh University of Medical Sciences, Torbat Heydariyeh, Iran

**Keywords:** Human papillomavirus, HPV vaccine, E7 oncoprotein, Stimulator of interferon genes, STING, CpG ODN 2395, Cyclic dinucleotide (CDN)

## Abstract

Persistent infection with high-risk genotypes of human papillomavirus (HPV) is the leading cause of cervical cancer. The HPV oncoprotein E7 is constitutively expressed in cervical cancer and considered as an essential target for tumor-specific immunity. The goal of this study was to develop a candidate therapeutic vaccine based on the mutated E7 protein that had possibly reduced transformation capacity while was able to elicit a robust immune response. Therefore, the mutant type of HPV 16 E7 (E7GRG) protein was recombinantly expressed in *E. coli*. The protein was then purified and formulated with 2’-3’cGAMP CDN and/or CpG-C ODN adjuvants and subcutaneously injected to female C57BL/6 mice. To evaluate the immunogenic response, lymphocyte proliferation, secretion levels of IFN-γ and IL-4 cytokines, granzyme B level, and total IgG and subclasses of IgG antibody were measured. The anti-tumor activity was evaluated in tumor-harboring C57BL/6 mice. The highest rate of cell proliferation, IFN-γ and granzyme B levels, and amount of IgG antibody were found in mice group that were injected by E7GRG + 2′-3′cGAMP + CpG-C. Therapeutic immunization with E7GRG + 2′-3′cGAMP + CpG-C also significantly suppressed TC-1 tumor growth in mice. In conclusion, the results demonstrated that E7GRG + 2′-3′cGAMP + CpG-C induced strong cell-mediated and humoral immune responses that resulted in inhibition of tumor in mouse model.

## Introduction

Cervical cancer is the fourth most common cancer among women worldwide as 569,847 new cases and 311,365 new deaths had been reported by the International Agency for Research on Cancer (IARC) in 2018 [1]. Human papillomavirus (HPV) is well-known as the etiological agent of cervical cancer. While more than 40 HPV types can infect the anogenital tract, 14 HPV types, designated as the high-risk HPV types including 16, 18, 31, 33, 35, 39, 45, 51, 52, 56, 58, 59, 68 and 73, are associated with cervical cancer development [2–5]. Among high-risk HPV types, HPV 16 and 18 are the leading cause and responsible for almost 71 % of cervical cancer globally [6].

Three prophylactic HPV vaccines, including Cervarix®, Gardasil®, and Gardasil® 9, are available for prevention of HPV infection and consequently HPV-related cancers [7–9]. It is documented that HPV vaccines are safe, effective and provide long-lasting protection against the HPV infections [10, 11]. However, these vaccines are unable to clear already established infections and do not mount the cell-mediated immune responses required to eliminate pre-existing lesions and even malignant tumors. Regarding sub-optimal prophylactic HPV vaccination program in the world, HPV infections and subsequent development of HPV-related cancers will continue to be a public health problem. Consequently, the development of therapeutic HPV vaccines seems to be of particular importance to treat HPV-associated cancers, particularly cervical cancer [12, 13].

Therapeutic vaccine strategies intend to combat precancerous or cancerous lesions by triggering cell-mediated immunity against HPV-infected cells. A therapeutic HPV vaccine should be directed against HPV antigens that are constitutively expressed in the infected cells and cancer cells. [14, 15]. The HPV E6 and E7 proteins are ideal target antigens since they are exclusively expressed in infected cells and consequently lead to transformation by disrupting the regulation of cell cycle [5, 16]. Current platforms of therapeutic HPV vaccines include viral-vector, bacterial-vector, peptide or protein-, nucleic acid (DNA and RNA), dendritic cell-based, and tumor cell-based vaccines; among which protein-based vaccines are a suitable candidate to treat HPV-associated cancers. The advantages of protein-based vaccines include having abundant CD4^+^ and CD8^+^ T cell epitopes, no HLA restrictions, easy to produce, high stability, and safety [12, 15]. However, protein-based vaccines are not very immunogenic, and co-administration of adjuvants is needed to elicit strong immune responses [17, 18].

An agonist of the intracellular stimulator of interferon genes (STING) pathway, cyclic di-nucleotide a 2′3′-cGAMP (CDNs), has been applied as a vaccine adjuvant in experiments [19]. It has been shown that CDNs induce anti-tumor responses *in vivo* through the signaling pathway stimulated by activation of STING that results in production of IFN-I and other cytokines essential for innate immunity [20]. Moreover, using of synthetic oligodeoxynucleotides (ODNs) containing unmethylated CpG motifs (CpG-ODNs) as an adjuvant can induce robust immune responses through activation of Toll-like receptor 9 (TLR9), leading to induction of IFNs and pro-inflammatory cytokines production, activation of natural killer cells, and elicitation of Th1-dominant immune responses [21, 22]. Overally, CpG ODNs as an adjuvant can improve the function of professional antigen-presenting cells and boost the generation of humoral and cellular vaccine-specific immune responses [22].

In this study a mutated form of E7 protein of HPV 16 with regard to reduced capacity for transformation was investigated as a therapeutic vaccine candidate. The mutations were previously predicted as not affecting the antigenic epitopes, and the protein was able to induce robust immune responses similar to the wild type protein [23]. Briefly, all potential mutants were evaluated for binding of peptide epitopes to MHC-I molecules, efficient processing by the proteasome system, and higher immunogenicity level of peptide epitopes. Finally, the mutated form of E7 protein that contained substantial substitution at positions C24G, L67R, and C91G (E7GRG) was considered as an appropriate candidate vaccine antigen, and upon formulation with 2′3′-cGAMP STING activator and CpG-C (ODN-2395) adjuvants, induced a potent immune response and inhibited tumor growth in mouse model.

## Materials and Methods

### Expression and purification of HPV 16 E7GRG protein

Synthesized HPV 16 E7GRG mutant sequence was digested by restriction enzymes *Eco*RI and *Sal*I and subcloned into pET-28a (+) vector (Novagen, USA) that added an N-terminal His-tag to the expressed protein. This vector was transformed into *Escherichia coli* BL21 strain (DE3) and was confirmed by PCR and restriction enzyme digestion. The transformed BL21 cell was incubated in LB broth (containing 50 mg/ml of kanamycin) at 37° C until the cell density reached OD_600_ nm of 0.8 to 1. The expression of E7GRG protein was induced in BL21 cells for 4 h with 0.2 mM isopropyl-β-D-thio-galactoside (IPTG), and the protein identity was confirmed by SDS-PAGE and Western blot analysis using His-Probe Antibody (H-3) (Santa Cruz Biotechnology, USA). Purification of the expressed protein was performed under denaturing condition using Ni/NTA column. For protein refolding, the HPV 16 E7GRG mutant protein was dialyzed three times at 4 ˚C with PBS containing 5 M urea, 2.5 M urea, and 0 M urea consecutively using dialysis membrane with 6–8 kDa cut-off. The concentration of the total protein was detected by Bradford assay.

### Animal experiments

To evaluate the induction of immune responses by purified HPV 16 E7GRG mutant protein, female C57BL/6 mice, 6–8 weeks of age, were randomly divided into eight groups (in groups 1–5, 5 mice and in groups 6–8, 3 mice were included). Groups 1 to 8 received subcutaneous injections as follow: group 1 was injected with 10 µg of purified protein without any adjuvants (E7GRG); group 2 was injected with 10 µg of purified protein combined with 10 µg of CpG-C ODN 2395 (E7GRG + CpG-C); group 3 received 10 µg of purified protein in combination with 4 µg of 2′-3′cGAMP (E7GRG + 2′-3′cGAMP); group 4 was injected with 10 µg of purified protein co-administered with 4 µg of 2′-3′cGAMP + 10 µg of CpG-C (E7GRG + 2′-3′cGAMP + CpG-C); groups 5 to 7 received 10 µg of CpG-C, 4 µg of 2′-3′cGAMP, and 4 µg of 2′-3′cGAMP + 10 µg of CpG-C, respectively; and group 8 received PBS. The immunization schedule was based on a three-dose regimen, where the booster immunizations were administered at 14 and 28 days after the primary injection. Blood samples were obtained from the retro-orbital vein seven days after the last administration, and the mice were then sacrificed according to established guidelines. The spleen of mice were harvested to prepare a single-cell suspension of each mouse. The experiments were approved by the Local Ethics Committee of Tehran University of Medical Sciences (Grant no. 34,892). All experiments were performed according to the Helsinki guidelines.

### BrdU lymphocyte proliferation assay

To measure cell proliferation via BrdU assay, the spleen cells of each mouse were subjected to 5-bromo-2’-deoxyuridine (BrdU) and were assessed with anti-BrdU Ab using indirect ELISA according to the manufacturer’s instruction of the commercial kit (Roche, Germany). Concisely, the spleen cells of each mouse were cultured in flat 96-well plates in triplicate (2 × 10^5^ cells per well). Then, 2 µg/ml of E7-specific CTL epitope (E7, amino acids 49–57) was added and incubated at 37°, 5 % CO_2_. The positive and negative controls were Concanavalin A (5 µg/mL) and the untreated cells in RPMI-1640 medium, respectively. After 72 h incubation, BrdU was added to each well and plates were incubated at 37 °C, 5 % CO_2_ for 24 h. Then, the culture medium was removed, cells were fixed, and DNA was denatured by adding fixDenat solution. Anti-BrdU-POD was added and followed by substrate reaction. Finally, the plates were read at OD_450_ nm using ELISA reader.

### IFN-γ and IL-4 detection by ELISPOT

Murine IFN-γ and IL-4 cytokines were measured by ELISpot kit (Mabtech, Sweden) according to the manufacturer’s instruction. Briefly, 2.5 × 10^5^ cells/well were pipetted directly into each well of the plate pre-coated with the IFN-γ or IL-4 capture antibody. The spleen cells were stimulated with 2 µg/ml of the HPV 16 E7 peptide (amino acids 49–57). Positive and negative wells were stimulated with 5 µg/ml Concanavalin A and culture medium, respectively. The plate was then placed in a humidified incubator at 37 °C with 5 % CO_2_ for 24 h. Following wash steps and incubation with a biotinylated detection antibody, Streptavidin-HRP conjugate was added followed by treatment with TMB substrate solution. After stopping color development, the number of spots that were correlated to the cells producing antigen-specific murine IFN-γ and IL-4 cytokines were counted using a stereomicroscope. The test was performed in triplicate for each mouse.

### Granzyme B release assay

Mouse granzyme B was measured by an ELISpot kit (R&D BioSystems, USA) according to the manufacturer’s instruction. In brief, 5 × 10^4^ cells/well were seeded in antibody-coated wells in triplicate and stimulated by 2 µg/ml of E7 (amino acids 49–57) peptide. The plates were incubated for 24 h at 37 °C, 5 % CO_2_. Following wash steps and incubation with a biotinylated detection antibody, alkaline-phosphatase conjugated streptavidin was added. Unbound enzyme was subsequently removed by washing and BCIP/NBT substrate solution was added. The blue-black colored precipitates formed at the site of cytokine localization, and appeared as spots, with each individual spot representing an individual mouse granzyme B secreting cell, were counted manually using a stereomicroscope.

### Measurement of total IgG and subclasses of IgG1 and IgG2a by ELISA

ELISA was used to determine the total IgG and IgG1 and IgG2a subclasses in the sera of immunized mice. To this end, Nunc-Immuno F8 MaxiSorp plate (Thermo Scientific, USA) was coated with 1 µg E7GRG protein in PBS at 4 °C overnight, blocked with 5 % skimmed milk, and incubated with 100 µl of 1:250 dilution of heat-inactivated mouse sera and incubated at 37 °C for 1 h. After wash steps with PBS/0.1 % Tween-20, wells were incubated for 1 h at 37 °C with HRP-conjugated goat anti-mouse (anti-IgG, IgG1 and IgG2a) antibodies at dilution of 1:4000 (Southern Biotech, USA). Reactions were developed with TMB solution, stopped with 2 N sulfuric acid, and absorbance was read at 450 nm. The reactions were determined positive as exceeding the mean absorbance ± 2 standard deviations (SD) of equal dilutions of control.

### ***In vivo*tumor inhibition assay**

TC-1 is a tumorigenic cell line which has been derived from primary lung epithelial cells of C57BL/6 mice and has been co-transformed with the E6 and E7 oncoproteins of HPV 16. The TC-1 cells were expanded in RPMI-1640 medium supplemented with 10 % FBS, 1 % penicillin/streptomycin, and 400 µg/ml G418 for two weeks, and were washed two times with PBS before injection. To perform tumor cell growth inhibition assay, C57BL/6 mice were subcutaneously injected with 2 × 10^5^ TC-1 cells suspended in PBS at the right shaved flank. After two weeks, when tumor mass was palpable, mice were subcutaneously injected by the different regiments of purified HPV 16 E7GRG mutant protein. The mice were put into 5 groups (each group include 5 mice) and received subcutaneous injections as follows: group 1 was injected with 10 µg of purified protein without any adjuvants (E7GRG); group 2 was injected with 10 µg of purified protein in combination with 10 µg of CpG-C (E7GRG + CpG-C); group 3 received 10 µg of purified protein combined with 4 µg of 2′-3′cGAMP (E7GRG + 2′-3′cGAMP); group 4 was injected with 10 µg of purified protein co-administered with 4 µg of 2′-3′cGAMP + 10 µg of CpG-C (E7GRG + 2′-3′cGAMP + CpG-C); and groups 5 received 10 µg of wild-type HPV 16 E7 protein combined with 4 µg of 2′-3′cGAMP + 10 µg of CpG-C (E7GRG + 2′-3′cGAMP + CpG-C). The immunization schedule was based on a two-dose regimen, where the booster immunizations were administered at seven days after the primary injection. Tumor size was measured twice a week using caliper, and determined using the *Carlsson* formula. Mice were sacrificed according to established guidelines when the largest tumor reached an ethical point.

### Statistical analysis

Data were expressed as mean ± standard deviation. Statistical analyses were carried out by Student’s *t*-test and Two-way/One-way ANOVA, followed by HSD-Tukey multiple comparisons test as appropriate. Data were analyzed using GraphPad Prism version 7.03 (GraphPad Software, USA). The *P*-value < 0.05 was considered statistically significant.

## Results

### Expression of HPV 16 E7GRG protein

Expression of HPV 16 E7GRG protein was performed in *E.coli* BL21 (DE3) strain and purified on Ni/NTA column. The E7GRG protein (17 KDa) was run on a 12 % SDS-polyacrylamide gel and was confirmed by the Western blotting using anti-His Ab.

### Animal experiment

To compare the immunogenicity of the protein, female C57BL/6 mice were immunized subcutaneously with three doses of antigens with or without 2′-3′cGAMP and/or CpG-C adjuvants in eight mice groups (Group 1: E7GRG; group 2: E7GRG + CpG-C; group 3: E7GRG + 2′-3′cGAMP; group 4: E7GRG + 2′-3′cGAMP + CpG-C; group 5: CpG-C, group 6: 5 2′-3′cGAMP; group 7: 2′-3′cGAMP + CpG-C; and group 8: PBS) at 0, 14, and 28 days. Immunized and control mice were daily monitored during the immunization and did not show any clinical signs of toxicity. Spleen lymphocyte collected from mice analyzed by lymphocyte proliferation response and the levels of IFN-γ, IL-4, granzyme B were measured. The sera collected from mice was evaluated by ELISA to measure antibody response.

To perform tumor cell growth inhibition assay, C57BL/6 mice were subcutaneously injected with TC-1 cells. After two weeks, when tumor mass was palpable, mice were subcutaneously injected with two doses of antigens with or without 2′-3′cGAMP and/or CpG-C adjuvants in five different groups (group 1: E7GRG; group 2: E7GRG + CpG-C; group 3: E7GRG + 2′-3′cGAMP; group 4: E7GRG + 2′-3′cGAMP + CpG-C; and groups 5: wild type E7GRG + 2′-3′cGAMP + CpG-C) at 0 and 7 days. The tumor size was measured up to one month after the immunization.

### Lymphocyte proliferation response

**Fig. 1:**
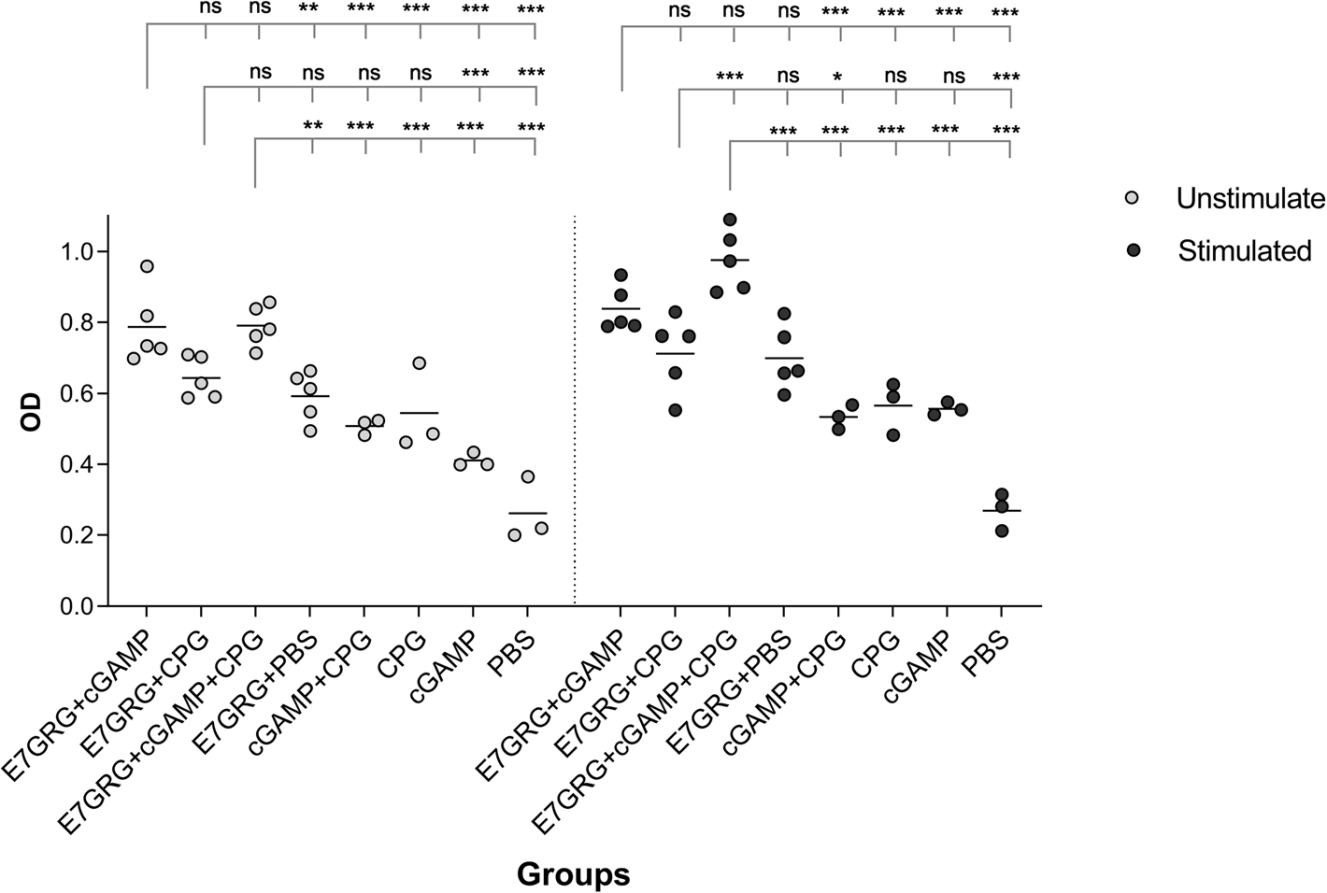
Lymphocytes proliferation response levels at 7 days after the last immunization in mice that were stimulated. (*p* < 0.05: *, *p* < 0.01: **, *p* < 0.001: *** and *p* < 0.0001: ****)

The results of spleen lymphocyte proliferation assay were shown in Fig. 1. It was shown that when both 2′-3′cGAMP and CpG-C adjuvants were simultaneously administered with mutant E7 protein (E7GRG + 2′-3′cGAMP + CpG-C), lymphocyte proliferative responses were at the highest rate compared to other groups and the difference reached a statistically significant level (*p* < 0.001 or *p* < 0.0001). It was also found that E7GRG + 2′-3′cGAMP + CpG-C promoted more strong lymphocyte proliferation response than other test groups (E7GRG + 2′-3′cGAMP and E7GRG + CpG-C), as the difference between E7GRG + 2′-3′cGAMP + CpG-C and E7GRG + CpG-C groups showed a statistically significant difference (*p* < 0.01). However, the difference between E7GRG + 2′-3′cGAMP + CpG-C and E7GRG + 2′-3′cGAMP was not reached statistically significant level.

Regard to stimulation status, our findings showed that only in immunized mice with E7GRG + 2′-3′cGAMP + CpG-C a significant difference was found before and after stimulation (*p* < 0.001), and no difference was found for other groups.

### The levels of IFN-γ and IL-4 cytokines

**Fig. 2:**
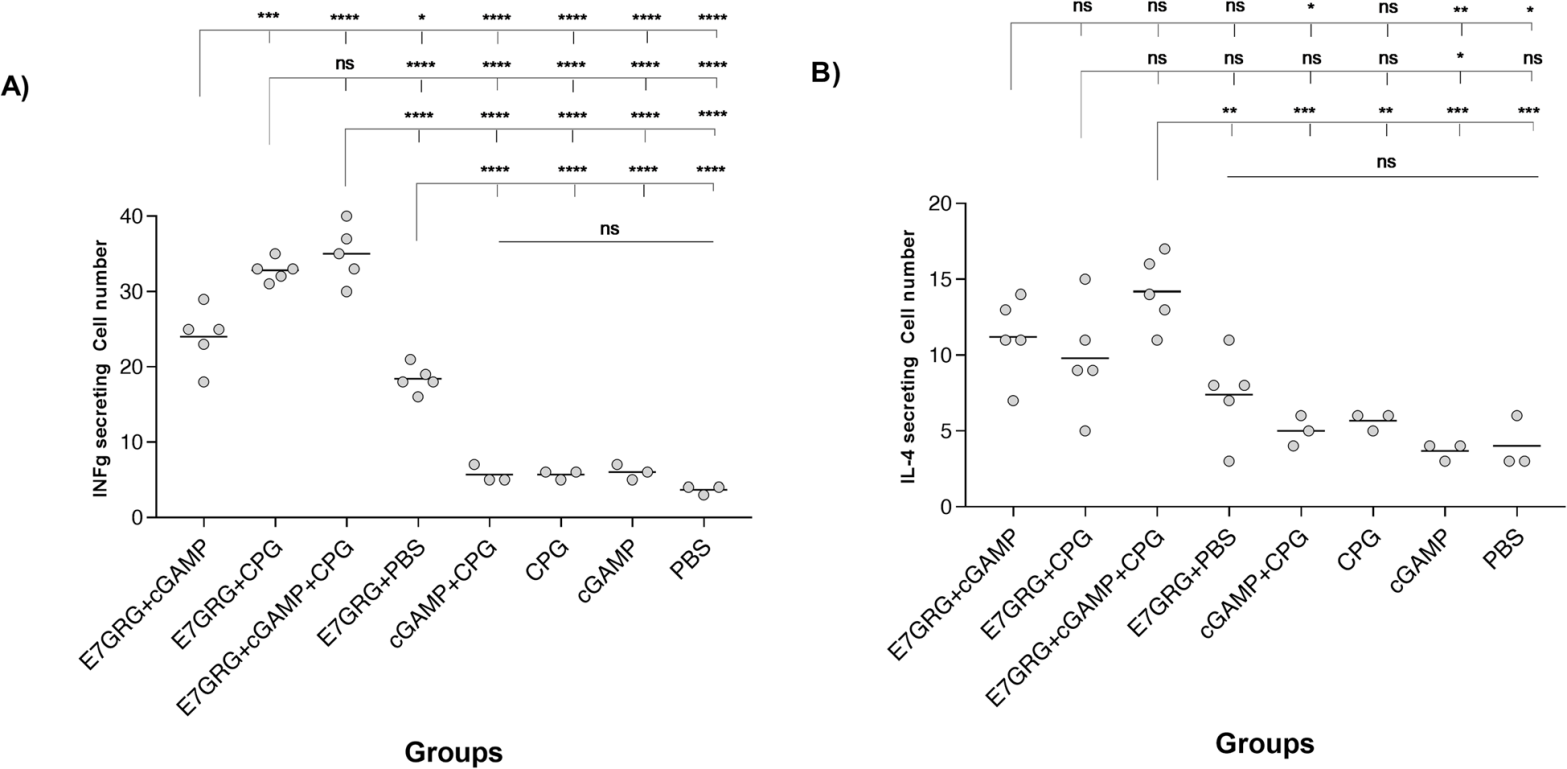
**a** IFN-γ level; and **b** IL-4 level at 7 days after the last immunization. IFN-γ and IL-4 concentrations were assessed in the supernatant of splenocytes of mice per group that were stimulated. Data is presented as spots per well. (*p* < 0.05: *, *p* < 0.01: **, *p* < 0.001: ***, *p* < 0.0001: **** and non-significant: ns)

The IFN-γ secretion levels of all test groups (E7GRG + 2′-3′cGAMP, E7GRG + CpG-C, and E7GRG + 2′-3′cGAMP + CpG-C) were significantly higher than the control groups (E7GRG + PBS, 2′-3′cGAMP, CpG-C, 2′-3′cGAMP + CpG-C, and PBS) and the difference was statistically significant (*p* < 0.0001) (Fig. 2). The highest rate of the IFN-γ level was found in E7GRG + 2′-3′cGAMP + CpG-C and E7GRG + CpG-C groups. IFN-γ levels in the supernatants of spleen cells from vaccinated mice with E7GRG + 2′-3′cGAMP + CpG-C and E7GRG + 2′-3′cGAMP showed a statistically significant difference (*p* < 0.0001) when re-stimulated with HPV E7 epitope (amino acids 49–57). However, a statistically significant difference was not observed among control groups.

Analysis of IL-4 levels revealed that there was a statistically significant difference between E7GRG + 2′-3′cGAMP + CpG-C and control groups (E7GRG + PBS, 2′-3′cGAMP, CpG-C, 2′-3′cGAMP + CpG-C, and PBS). As indicated in Fig. 2, no statistically significant difference was observed among control groups.

Our finding indicated that the E7GRG + 2′-3′cGAMP + CpG-C regimen predominantly elicited a Th1 cytokine profile and induced CD8^+^ T-cell responses.

### The level of granzyme B

**Fig. 3:**
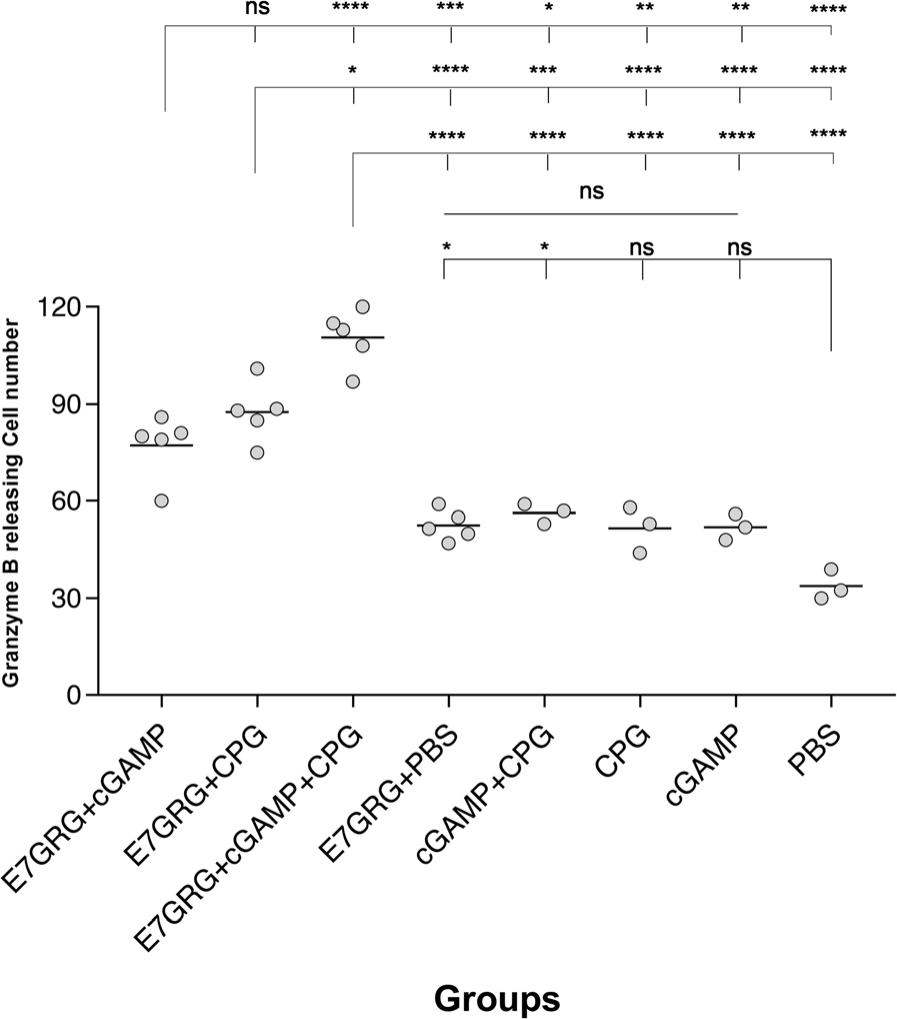
Granzyme B level at 7 days after the last immunization. Granzyme B concentration was evaluated in splenocytes of mice per group that were stimulated. Data is presented as spots per well. (*p* < 0.05: *, *p* < 0.01: **, *p* < 0.001: ***, *p* < 0.0001: **** and non-significant: ns)

The levels of granzyme B indicated a statistically significant difference in all three test groups (E7GRG + 2′-3′cGAMP, E7GRG + CpG-C, and E7GRG + 2′-3′cGAMP + CpG-C) compared to control groups (E7GRG + PBS, 2′-3′cGAMP, CpG-C, 2′-3′cGAMP + CpG-C, and PBS) (Fig. 3). Among test groups, mice that received E7GRG + 2′-3′cGAMP + CpG-C regimen presented an upregulated level of granzyme B when stimulated with the HPV 16 E7 epitope (amino acids 49–57), and the difference between E7GRG + 2′-3′cGAMP + CpG-C and E7GRG + 2′-3′cGAMP or E7GRG + CpG-C reached a statistically significant level (*p* < 0.0001 and *p* < 0.05, respectively) (Fig. 3). Our results revealed that the highest rate of granzyme B levels was produced by E7GRG + 2′-3′cGAMP + CpG-C regimen.

### Antibody responses

**Fig. 4:**
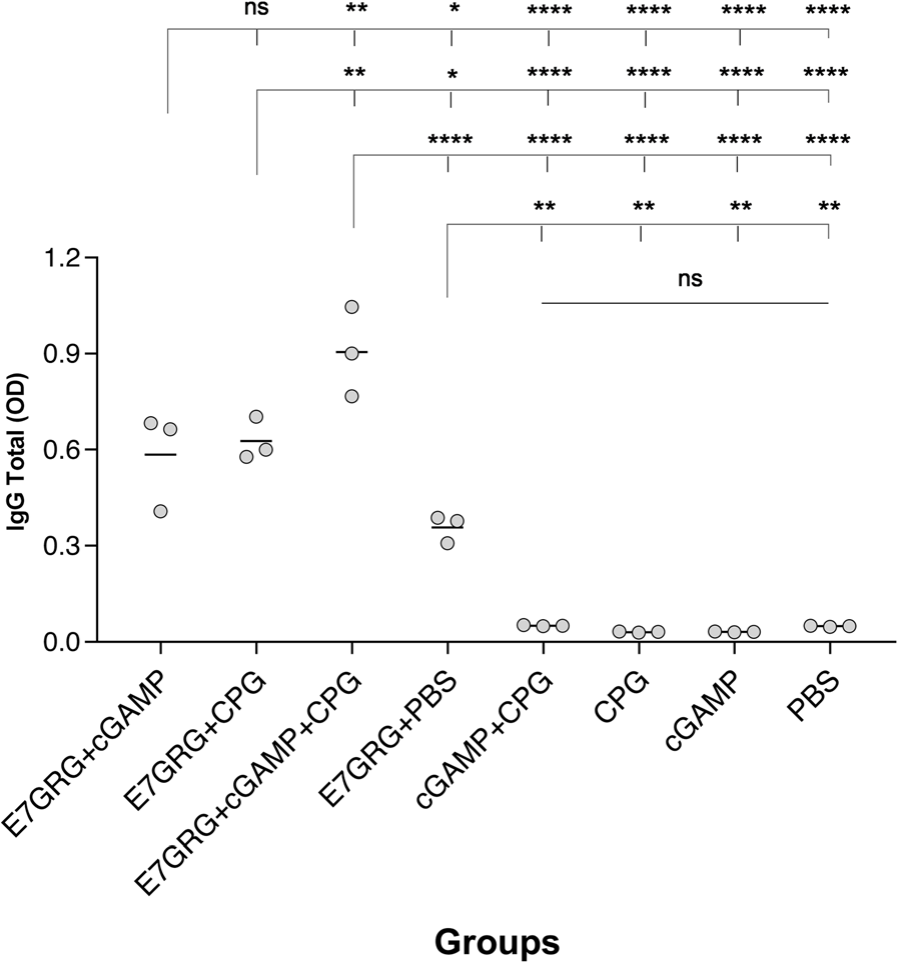
Total IgG antibody against E7GRG protein in serum samples of C57BL/6 mice immunized subcutaneously at 7 days after the last immunization. (*p* < 0.05: *, *p* < 0.01: **, *p* < 0.001: ***, *p* < 0.0001: **** and non-significant: ns)

Our results showed that E7GRG + 2′-3′cGAMP + CpG-C induced more total IgG antibody response compared to other test groups (E7GRG + 2′-3′cGAMP and E7GRG + CpG-C) (*p* < 0.01) and control groups (E7GRG + PBS, 2′-3′cGAMP, CpG-C, 2′-3′cGAMP + CpG-C, and PBS) (*p* < 0.0001) (Fig. 4). However, no significant difference was observed among control groups. As shown in Fig. 4, the difference between E7GRG + 2′-3′cGAMP group or E7GRG + CpG-C group with each control groups were statistically significant. Interestingly, E7GRG without any adjuvants could evoked the production of total IgG and the difference between E7GRG + PBS with 2′-3′cGAMP, CpG-C, 2′-3′cGAMP + CpG-C, and PBS groups reached a statistically significant level (*p* < 0.01).

**Fig. 5:**
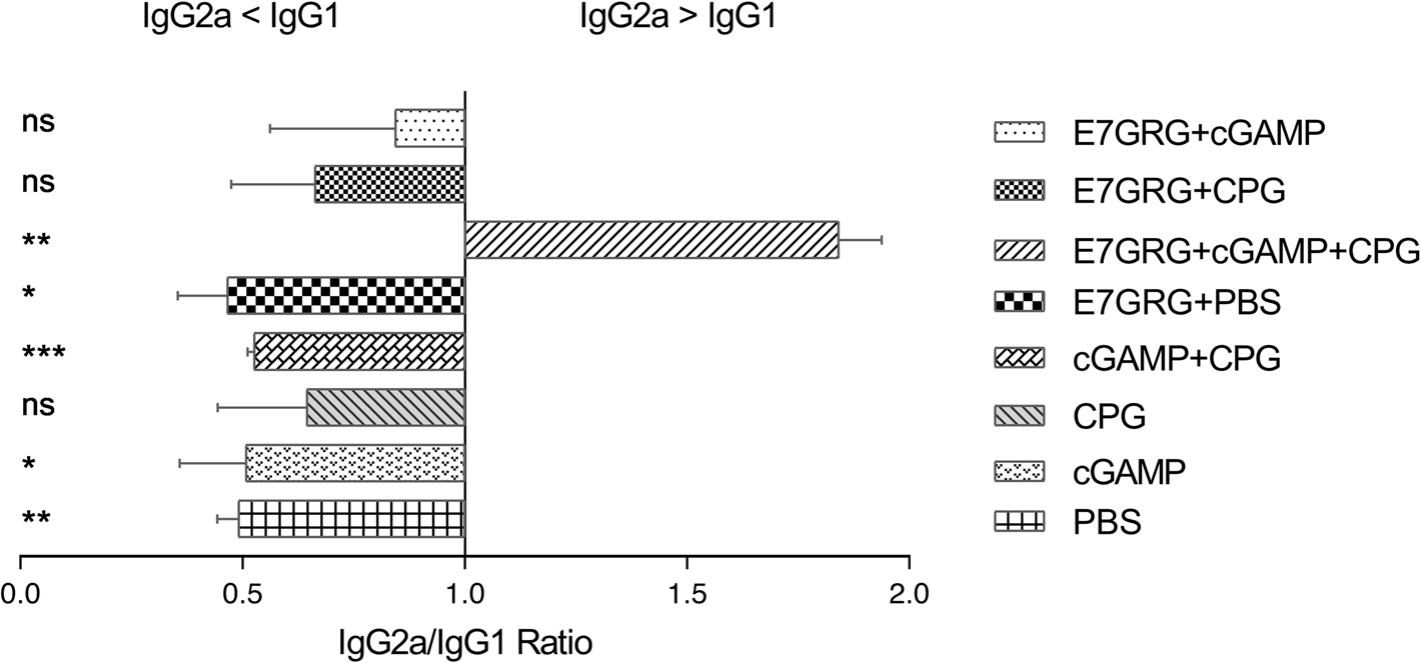
The ratio of IgG2a /IgG1 antibody against E7GRG protein in serum samples of C57BL/6 mice immunized subcutaneously at 7 days after the last immunization. (*p* < 0.05: *, *p* < 0.01: **, *p* < 0.001: ***, *p* < 0.0001: **** and non-significant: ns)

As indicated in Fig. 5, evaluation of IgG antibody subclasses indicated that the immunization of mice with E7GRG + 2′-3′cGAMP + CpG-C induced higher IgG2a antibody responses. The increased IgG2a/IgG1 ratio in mice immunized with E7GRG + 2′-3′cGAMP + CpG-C indicated a tendency to Th1-type immune response.

### ***In vivo*tumor growth suppression assay**

**Fig. 6:**
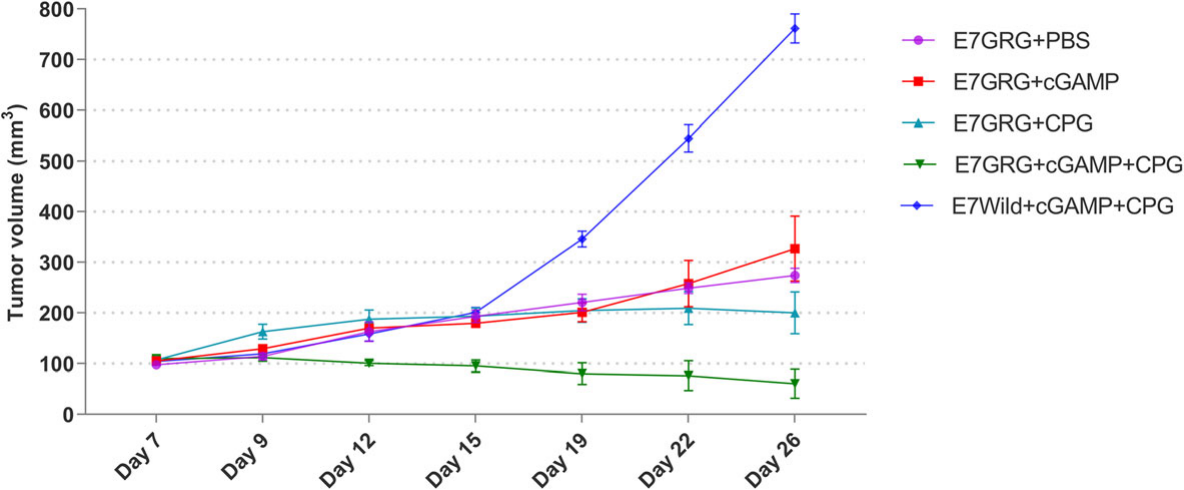
TC-1 cell growth inhibition by E7GRG protein in tumor bearing mice. Data presented as mean ± SEM of tumor sizes

In mice that were injected with TC-1 tumor cells, the tumor size was measured before immunization up to one month after the immunization. The results showed the mice that received E7GRG + 2′-3′cGAMP + CpG-C significantly suppressed tumor growth as compared to other test groups (E7GRG + 2′-3′cGAMP and E7GRG + CpG-C) (*p* < 0.05) and control groups (WildE7 + 2′-3′cGAMP + CpG-C) (*p* < 0.0001) (Fig. 6). Interestingly, a significant increase in the size of tumor was found in control group that received the wild type E7 protein along with adjuvants 2′-3′cGAMP + CpG-C (Fig. 6).

Our findings showed that, as a therapeutic model, immunization with E7GRG + 2′-3′cGAMP + CpG-C inhibited TC-1 tumors growth in mice. It seems that the combination of two adjuvants (2′-3′cGAMP and CpG-C) had a synergistic effect that led to tumor growth suppression.

## Discussion

Therapeutic vaccine studies are growing up because of the necessity of developing new methods for anti-tumor therapy as standard protocols in clinical trials [24]. Furthermore, recent advances in therapeutic methods take advantage of our knowledge on how the immune system encounters with viral infections [25], and that eliminate lesions and malignant tumors through cell-mediated immune responses against HPV-infected cells [17]. Several therapeutic HPV vaccines are in different clinical trial phases in attempt to treat HPV-associated cancers [15].

In this study, the HPV 16 E7 protein-containing C24G, L67R, and C91G mutations were expressed in *E. coli* BL21 starin (DE3). It is well-documented that specific mutations within some E7 domains disrupt its transforming ability, e.g., deletion of the amino acids 6 to 10 in conserved region 1 (CR1); substitution of C to G at positions 24, 58 and 91 [26]. It has been found that while these mutations on HPV 16 E7 protein result in destroying transformation activity, they do not affect the immunogenicity of the protein. Consequently, it seems that mutated form of E7 protein can be used as a therapeutic vaccine antigen as a safer alternative rather than wild-type E7 protein [27].

Although therapeutic protein vaccines are considered safe, they are weakly immunogenic and adjuvants are mostly needed for enhancing their immunogenicity [17]. It has been shown that E7 protein formulated with QuilA or CpG oligonucleotides induces CD8^+^ T cell responses. Indeed, co-administration of the HPV 16 E7 protein with CpG adjuvant in tumor mouse model resulted in regression of tumor due to evoking of HPV16-specific cytotoxic T-lymphocyte responses [28].

In the current study, the immunogenicity of HPV 16 E7GRG protein with different adjuvant formulations, including 2′- 3′cGAMP, CpG-C (ODN 2395), and 2′- 3′cGAMP + CpG-C was evaluated. The results showed that E7GRG + 2′-3′cGAMP + CpG-C elicited a strong cell-mediate immunity as the highest rate of lymphocyte proliferation and IFN-γ and granzyme B levels were shown in this group, and the difference between this group and each of other groups was statistically significant. It is known that an effective HPV therapeutic vaccine should activate a potent cell-mediated immune response where CD4^+^ T cells support CD8^+^ T cells by secreting cytokines such as IL-2 and IFN-γ [29]. Granzyme B also has an essential role in induction of apoptosis in virus-infected cells [30], and CTLs and NK cells use perforin/granzyme cytotoxic pathway to eliminate virus-infected cells and tumors [19, 25, 31]. The results of humoral immune responses, regarding the IgG2a/IgG1 ratio, was also indicated a shift towards the Th1-type immune response.

Several E7-based protein vaccines have been developed and examined in animal model. Indeed, fusion protein consisting of the HPV 16 E7 and *Mycobacterium bovis* bacille Calmette–Guérin hsp65 could trigger CTL responses in mice that resulted in tumor regression [32]. A fusion protein comprising of the HPV 16 E7 and domains I and II of *Pseudomonas aeruginosa* exotoxin A while co-administered with either GPI-0100 or CpG adjuvants elicited robust HPV specific E7 CTL responses and protection from tumor challenge [33]. Another fusion protein vaccine candidate consisted of the HPV 16 E7 protein and a peptide derived from the Limulus polyphemus anti-lipopolysaccharide factor (LALF31–51). It was shown that vaccination with LALF(32–51)-E7 fusion protein induced T cell responses and inhibited the tumor growth [34].

In this study, 2′-3′cGAMP CDN was selected as a STING ligand. It has been shown that the STING pathway induces an anti-tumor immune response. Therefore, STING agonists are broadly developed as a new agent for cancer therapy [35]. Studies suggest that CDNs are potential adjuvants of vaccines that can induce antigen-specific B- and T-cell responses [36]. Furthermore, a recent study has shown that activation of the STING-TBK1-IRF3 pathway by the STING ligand, DMXAA, led to the induction of undesired type 2 immune responses [37]. Therefore, the therapeutic application of STING ligands in cancer immunotherapy might be limited. Interestingly, it has been demonstrated that a combination of the TLR9 ligand, K3 CpG, with the STING ligand, 2′- 3′cGAMP, induced a higher IFN-γ secretion and CTL activation. This combination resulted in a shift towards the Th1-type immune response. Moreover, this combination is a potent antigen-free anti-tumor agent that can induce the suppression of B16F10 and EG7 tumors in mice tumor models. Collectively, the administration of both K3 CpG and 2′- 3′cGAMP was more efficient than their single-use [38].

Inhibition of TC-1-induced tumors growth was investigated using the mouse model. Our findings showed that immunization with E7GRG protein formulated with either 2′-3′cGAMP or CpG-C elicited a moderately effective immune response against TC-1-induced tumors and caused suppression of tumor growth. However, HPV 16 E7GRG co-administered with 2′-3′cGAMP + CpG-C adjuvants significantly prolonged survival that was potentially associated with upregulation of E7-specific CTL and Th1 responses when compared to control-treated mice. These data suggest that using two adjuvants simultaneously had synergetic effect to elicit a potent cell-mediated immunity leading to inhibition of tumor growth. It has been shown that natural and synthetic agonists of nucleic acid-sensing signaling pathways can activate cell death in tumor cells and recruit immune cells including DCs, NK cells, and CD8^+^ T cells into the tumor microenvironment [39].

Interestingly, a significant increase in the size of tumor was found in control group that received the wild type E7 protein along with 2′-3′cGAMP + CpG-C adjuvants. Conversely, Petrone et al., have immunized mice with 1–3 doses of wild-type E7 protein without adjuvant and have shown 100 % tumor protection in triple dose and 40 % protection in double dose-injected mice after challenging with 1 × 10^5^ TC-1 cells [40]. Comparably, we used double dose of wild-type E7 protein (as the control group) formulated with 2′-3′cGAMP + CpG-C, and challenged the mice with 2 × 10^5^ TC-1 cells. The tumor protection after about four weeks was significantly lower than the group immunized with the mutant form of the protein formulated with the same adjuvants.

## Conclusions

The immunogenicity of mutant HPV 16 E7 protein (E7GRG) in combination with 2′- 3′cGAMP and CpG-C adjuvants was evaluated in mice. Our findings showed that this protein, as a therapeutic vaccine candidate antigen, could stimulate cellular and humoral immune responses and mediated tumor growth suppression. It was demonstrated that co-administration of the two 2′-3′cGAMP and CpG-C adjuvants had a synergistic effect to establish a shift towards the Th1 immune response, and leading to reduced tumor growth. Therefore, this vaccine formulation could be a promising therapeutic candidate vaccine for HPV 16 established infections and HPV-associated tumors.
